# Patterns and Variability of Projected Bioclimatic Habitat for *Pinus albicaulis* in the Greater Yellowstone Area

**DOI:** 10.1371/journal.pone.0111669

**Published:** 2014-11-05

**Authors:** Tony Chang, Andrew J. Hansen, Nathan Piekielek

**Affiliations:** Department of Ecology, Montana State University, Bozeman, Montana, United States of America; DOE Pacific Northwest National Laboratory, United States of America

## Abstract

Projected climate change at a regional level is expected to shift vegetation habitat distributions over the next century. For the sub-alpine species whitebark pine (*Pinus albicaulis*), warming temperatures may indirectly result in loss of suitable bioclimatic habitat, reducing its distribution within its historic range. This research focuses on understanding the patterns of spatiotemporal variability for future projected *P.albicaulis* suitable habitat in the Greater Yellowstone Area (GYA) through a bioclimatic envelope approach. Since intermodel variability from General Circulation Models (GCMs) lead to differing predictions regarding the magnitude and direction of modeled suitable habitat area, nine bias-corrected statistically down-scaled GCMs were utilized to understand the uncertainty associated with modeled projections. *P.albicaulis* was modeled using a Random Forests algorithm for the 1980–2010 climate period and showed strong presence/absence separations by summer maximum temperatures and springtime snowpack. Patterns of projected habitat change by the end of the century suggested a constant decrease in suitable climate area from the 2010 baseline for both Representative Concentration Pathways (RCPs) 8.5 and 4.5 climate forcing scenarios. Percent suitable climate area estimates ranged from 2–29% and 0.04–10% by 2099 for RCP 8.5 and 4.5 respectively. Habitat projections between GCMs displayed a decrease of variability over the 2010–2099 time period related to consistent warming above the 1910–2010 temperature normal after 2070 for all GCMs. A decreasing pattern of projected *P.albicaulis* suitable habitat area change was consistent across GCMs, despite strong differences in magnitude. Future ecological research in species distribution modeling should consider a full suite of GCM projections in the analysis to reduce extreme range contractions/expansions predictions. The results suggest that restoration strageties such as planting of seedlings and controlling competing vegetation may be necessary to maintain *P.albicaulis* in the GYA under the more extreme future climate scenarios.

## Introduction

Over the next century, it is expected that most of North America will experience climate changes related to increased concentrations of anthropogenic greenhouse gas emissions and natural variability [1]. At regional scales these changes are highly variable and can result in areas of increased mesic, xeric, or even hydric habitat conditions relative to present day. These shifting climates in turn also transform the suitable habitat for individual species that may result in changes in species composition and dominant vegetation types.

Whitebark pine (*Pinus albicaulis*) is a native conifer of the Western U.S. that is considered a keystone species in the sub-alpine environment. It provides a food source for animals such as the grizzly bear (*Ursus arctos*), red squirrel (*Tamiasciurus hudsonicus*), and Clark's nutcracker (*Nucifraga columbiana*) [2]. It also serves the ecosystem functions of stabilizing soil, moderating snow melt and runoff, and facilitating establishment for other species [2], [3]. Whitebark pine has experienced a notable decline in the past two decades within the U.S. Northern Rockies due to high rates of infestation from the mountain pine beetle (*Dendroctonus ponderosae*) and infections from white pine blister rust (*Cronartium ribicola*), resulting in an 80% mortality rate within the adult population [4]–[7]. Given the potential loss of important ecosystem functions that whitebark pine contribute to the landscape under this mortality event, there is an emphasis to understand the climate characteristics of its habitat to identify the restoration strategies and locations that may aid the persistence of the species under future climates.

One method of understanding species response to climate change is through bioclimate niche modeling, which has become a common practice for assessing potential vegetation shifts under new environmental conditions [8]–[13]. Ecological niche theory proposes there exists some range of bioclimatic conditions within which a species can persist [14]. In bioclimatic niche modeling, the realized niche is modeled by empirical relationships between the presence or absence of a species and the associated abiotic, and sometimes biotic, variables that describe the niche space. Bioclimatic models assume that species are in equilibrium with their environment and that the current abiotic relationships reflect a species environmental preferences which may be retained into the future [15], [16]. At macro scales, bioclimatic approaches have demonstrated success at predicting current distributions of species [17], [18]. Most bioclimatic models do not explicitly consider the many additional ecological factors that ultimately influence a species distribution such as dispersal, disturbance, or biotic interaction. Thus the approach does not predict where a species will actually occur in the future, but rather it predicts locations where climatic conditions will be suitable for the species.

Bioclimatic niche methodology has demonstrated utility in modeling historic ranges of species for conservation and management applications. By modeling the present day suitable habitat and then projecting those habitats into the future, bioclimatic niche models can serve as the first step filter for conservation action plans, such as mapping suitable species reintroduction sites or habitat reserve selection [19]–[21]. For *P.albicaulis*, McLane and Aitken [22] utilized bioclimate niche models to successfully implement experimental assisted migration on persisting climate habitat in British Columbia. Additionally, models of *Pinus flexis*, a closely related species of five needle pine, have been used to evaluate management options in Rocky Mountain National Park [23]. Given these examples, an effort to model and projected suitable climate habitat for *P.albicaulis* within a regional domain can provide valuable insight to land resource managers.

In this study, we present a bioclimatic habitat model for *P.albicaulis* within the Greater Yellowstone Area (GYA). Although *P. albicaulis* has a range-wide distribution that is split into two broad sections, one along Western North America: the British Columbia Coast Range, the Cascade Range, and the Sierra Nevada; and the other section in the Intermountain West that covers the Rocky Mountains from Wyoming to Alberta [2], [24]; the GYA was selected as the primary geographic modeling domain for three reasons: 1) evidence that the *P. albicaulis* sub-population in the GYA is genetically distinct from other regional populations with different climate tolerances [25]; 2) the high regional investment in *P. albicaulis* conservation in the area [6]; 3) the high density of climate stations within the region. Climate within the GYA is highly heterogenous due to complex topography, and sharp elevational gradients. Current knowledge of the region expects climate to shift towards increased mean annual temperatures and earlier spring snowmelt [26], [27]. This shift is expected to have an impact on the total suitable habitat area for *P. albicaulis*. Modeling at a regional scale can provide a finer resolution spatially explicit description of the bioclimatic envelope of *P. albicaulis* in the GYA.

**Figure 1:**
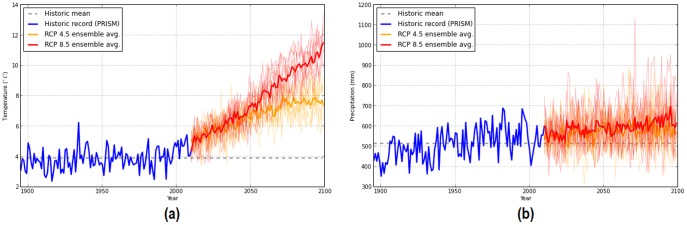
Historic and projected climates variables for the GYA from 1895–2099 under RCP 4.5 and 8.5 scenarios. Light shaded orange and red lines represent individual GCMs for RCP 4.5 and 8.5 respectively. Bold lines represent GCM ensemble average. (a) Mean annual temperature (b) Mean annual precipitation.

Here we also present an opportunity to investigate the effect of future climate variability on projected species distributions. In 2013, the World Climate Research Programme Coupled Model released the new generation General Circulation Model (GCM) projections through the Coupled Model Intercomparison Project Phase 5 (CMIP5) [28]. These new GCM projections also include four possible climate futures are modeled with each GCM under the Representative Concentration Pathways (RCP) of greenhouse gas/aerosol. These RCP scenarios designate four different levels of radiative forcing (2.6, 4.5, 6.0 and 8.5 W/m^2^) that may occur by the year 2099 [29]. In practice, research of future species suitable climate generally use a small suite of GCM/RCP combinations to project future climate [8], [11], [30]. However, internal variability in these GCMs that arise from modeled coupled interactions among the atmosphere, oceans, land, and cryosphere can result in atmospheric circulation fluctuations that are characteristic of a stochastic process [31]. Such intrinsic atmospheric circulation variations from model structure induce regional changes in air temperature and precipitation on the multi-decadal time scale [31]. For the GYA specifically, this GCM variability has be observed with mean annual temperatures projected to increase by 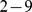
°C and mean annual precipitation to change by −50 to +225 mm (Fig. 1). This suggests that magnitude and direction of projected species distributions at a regional scale can vary depending on the GCM selected and the modeled species response to more xeric or mesic future climate conditions [32].

To summarize, this study presents a bioclimate niche model for *P. albicaulis* based on historic climate observations and field sampling of *P. albicaulis* presence and absence. Using this modeled bioclimate envelope, projections of future total climate suitable habitat area under nine GCMs and two RCP scenarios will be measured. Since different GCMs may project a diverging spectrum of climates, it is expected that measures of total suitable habitat will reduce with varying degrees of area loss. It is also expect that number and size of continous patches of *P. albicaulis* habitat will reduce due to the limited available number of sub-alpine areas distributed within the landscape. This research provides an analysis of the variability of biotic response under a large suite of GCMs to provide managers/researchers with a measure of the uncertainty associated with future species distribution models. Furthermore, this analysis explicitly describes the spatial patterns of bioclimatic niches for *P. albicaulis* to gain a better understanding of topographic characteristics, such as elevation, on suitable habitat. Changes in these spatial patterns are examined through quantifying landscape patch dynamic that may result from GCM projections to understand the species trends for persistence on the landscape.

## Methods

### Study area

**Figure 2:**
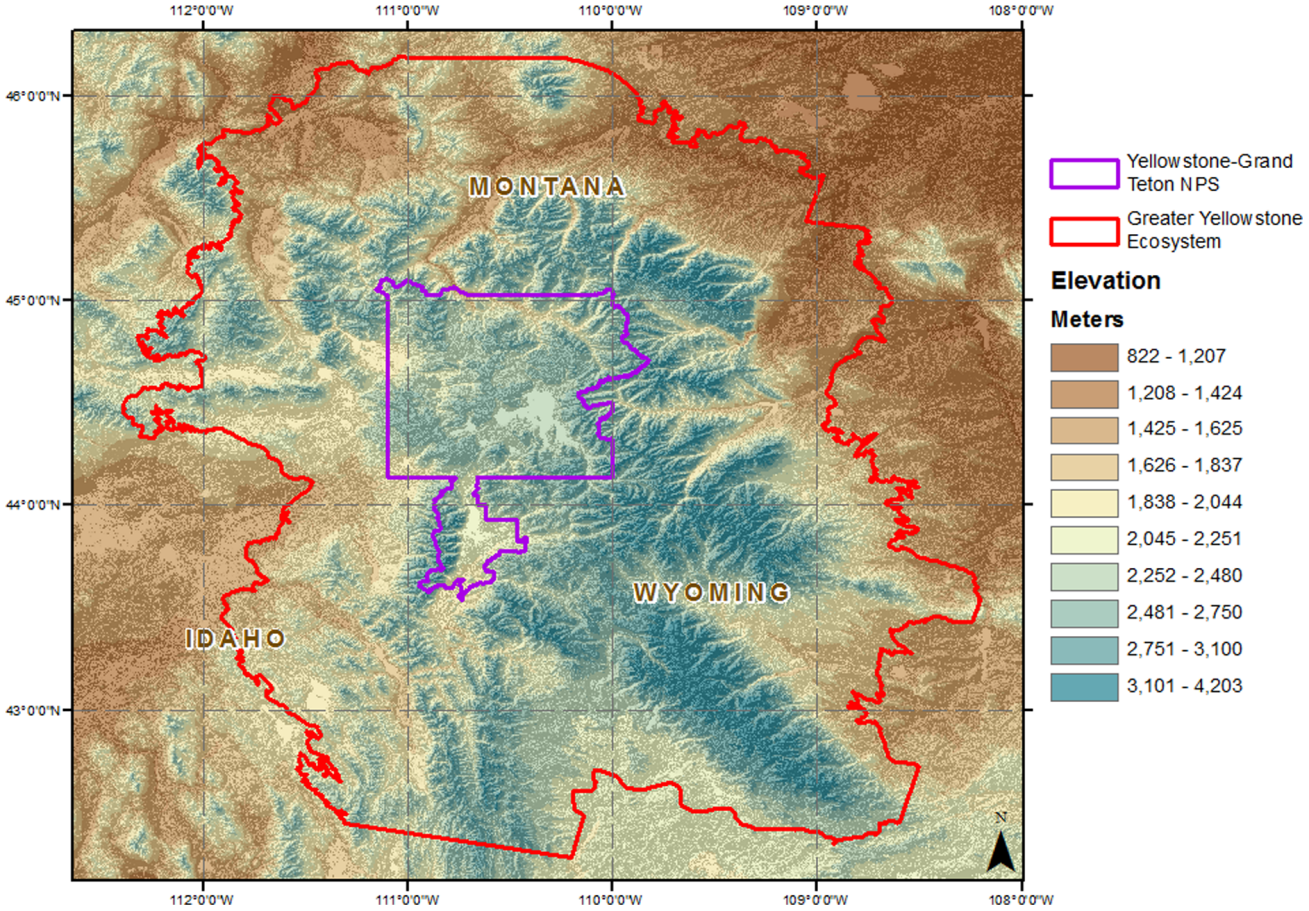
The Greater Yellowstone Area, representing an area of 150,700 km^2^ with an elevational gradient from 522–4,206 m.

The GYA, which includes Yellowstone National Park, Grand Teton National Park, and a number of state and federally managed forests, is a mid- to high-latitude region in the Northern Rocky Mountains of western North America. Conifers are dominant in the range, with forest types composed of *Pinus contorta*, *Abies lasiocarpa*, *Pseudotsuga menziesii*, *Pinus albicaulis*, *Juniperus scopulorum*, *Pinus flexis* and *Picea engelmannii*, although the deciduous hardwood *Populus tremuloides*, is also wide spread. Plateaus and lowlands are dominated by species of *Artemisia tridentata* and open grasslands of mixed composition. The GYA study area encompasses 150,700 km^2^ with an elevational gradient from 522–4,206 m that represents 14 surrounding mountain ranges (Fig. 2).

### Data

#### Biological data

Field observations of *P. albicaulis* adult presences and absences were compiled from three data sources. First, 2,545 observations from the Forest Inventory and Analysis (FIA) program were assembled. FIA plots are located on a regular gridded sampling design with one plot at approximately every 2,500 forested hectares, with swapped and fuzzed exact plot locations within 1.6 km to protect privacy [33]. Gibson et al. [34] found that model accuracy to not be dramatically affected by data fuzzing, but to provide the most spatial accuracy, this study culled FIA field points where measured elevation were 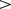
300 m different from a 30 m USGS DEM [35]. To capitalize on additional field observations of *P. albicaulis* within the study area, and because false absences are one of the most problematic data issues in constructing bioclimatic niche models [36]; supplementary points were drawn from the Whitebark/Limber Pine Information System (WLIS) [37], and long-term monitoring plots established by the National Park Service Greater Yellowstone Inventory and Monitoring Network (GYRN) [38]. The presences in these two additional datasets were collocated within predictor pixels of FIA absence to correct for false absences. In doing so, only one *P. albicaulis* presence or absence record was associated per predictor pixel, thereby avoiding issues associated with sampling bias that are common when building bioclimate niche models with data from targeted surveys [39]. This compilation of data represents an effort for “completeness” as described by Kadmon et al. [40] and Franklin [36], to capture all climate conditions where a species does exist. New data sources added 119 *P. albicaulis* presences that would have been missed by using FIA data alone, for a total of 938 presences and 1,633 absences.

“Adult” class *P. albicaulis* were selected for modeling based on a recorded diameter at breast height (DBH) 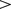
20 cm. *P. albicaulis* within the Central Montana are reported to reach 100 years of age at approximately 8–12 m in height with DBHs between 15–20 cm [41]. Given previous silvicultural studies, it was assumed that 20 cm DBH *P. albicaulis* represent adult class individuals for the GYA, with potential to reproduce [24]. Furthermore, this study focused on adult size class due to difficulties distinguishing younger age class *P. albicilus* from *P. flexis*.

#### Historic climate data

Climate inputs for modeling were acquired from the 30-arc-second (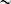
800 m) monthly Parameter-elevation Regressions on Independent Slopes Model (PRISM), a derived product that interpolates local station measurements across a continuous grid [42]. PRISM data includes monthly average minimum temperatures (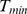), maximum temperature (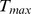), mean temperature (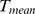), and mean precipitation (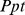). All monthly data were averaged for the temporal extent of 1950–1980 for bioclimatic niche model fitting. The 1950–1980 temporal extent was selected for modeling since: 1) a sufficient density of weather stations were operating by 1950 to provide a reasonable network; 2) evidence of anthropogenic warming that begins in the late 1980s; 3) trees old enough to bear seeds today likely established under a similar climates to the 1950–1980 period.

#### Water balance

A Thornthwaite-based dynamic water balance model was used to estimate a number of variables that include actual evapotranspiration (AET) and potential evapotranspiration (PET) [43]–[45]. The model required only monthly mean temperatures, dew point temperatures, and precipitation (see Text S1). Water was stored as soil moisture or in surface snowpack, with the excess taking the form of evaporated vapor or loss through seepage/runoff. In addition to the climatic variables, latitude and physical characteristics of the soil were required to define water holding capacity. Soil attributes assigned by the Soil Survey Geographic (STATSGO) datasets were allocated from the Natural Resource Conservation Service at a 30-arc-second resolution to determine soil water holding capacity and estimates for soil depth [46]. All water balance variables, which include PET, AET, soil moisture, vapor pressure deficit (vpd), and snow water equivalent (pack), were averaged by month over 1950–1980 to match with historic climate data for bioclimate model fitting.

#### GCM data

The general circulation model (GCM) experiments conducted under CMIP5 for the Intergovernmental Panel on Climate Change Fifth Assessment Report provided future projected climate data sets for assessing the effects of global climate change. Using a Bias-Correction Spatial Disaggregation (BCSD) approach, an archive of statistically down-scaled CMIP5 climate projections for the conterminous United States at 30-arc-second spatial resolution was assembled by the NASA Center for Climate Simulation NEX-DCP30 [47]. For this analysis, a subset of the total GCM models available from NASA were selected that best represent the Northwestern US. Rupp et al. [48] recently presented an analysis of GCM performance versus the observed historic climate in the U.S. Pacific Northwest under 18 specified climate metrics. In their analysis, Rupp et al. ranked GCMs for accuracy using an empirical orthogonal function (EOF) analysis of the total normalized error compared to reference data. This analysis selected models with a normalized error score 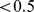 as a threshold to cull the full suite of GCMs to the top nine models. These GCMs were used to project modeled *P. albicaulis* distributions into the future (Table 1). Two RCP scenarios were selected to understand effects of differing carbon futures under climate change from 2010 to 2099. RCP 4.5 was the first, representing increased radiative forcing until stabilization of greenhouse emissions between 2040–2050 and total radiative forcing of 4.5 W/m^2^ by 2099. RCP 8.5 was the second, representing the “business as usual” scenario, with uncontrolled radiative forcing increasing with stabilization of 8.5 W/m^2^ by 2099 [49], [50].

**Table 1:** General Circulation Models for analysis.

Name	Institute	Country
CESM1-CAM5	National Center for Atmospheric Research	US
CCSM4	National Center for Atmospheric Research	US
CESM1-BGC	National Center for Atmospheric Research	US
CNRM-CM5	Centre National de Recherche Meteorologiques	FR
HadGEM2-AO	Met Office Hadley Centre Climate Programme	UK
HadGEM2-ES	Met Office Hadley Centre Climate Programme	UK
HadGEM2-CC	Met Office Hadley Centre Climate Programme	UK
CMCC-CM	Centro Euro-Mediterraneo per Cambiamenti Climatici	ITA
CanESM2	Canadian Centre for Climate Modelling and Analysis	CAN

Selection of AR5 GCMs that represent historic climate in the U.S. Pacific Northwest region for future bioclimate habitat modeling.

### Modeling methods

A random forest (RF) [51] algorithm was used to create a bioclimate niche model of *P. albicaulis* in the GYA. Random forest is an ensemble learning technique that generates independent random classification trees using a subset of the total predictor variables and classifies a bootstrap random subsample of the data. These trees are aggregated and a majority vote over all trees in the random forest defines the resulting response class. This method of random trees with subsampling ensures a robust ensemble classification reducing overfitting and collinearity issues, especially with a large number of trees [9], [51]–[53]. The python programming language (Python 3.3) and the Scikit-Learn library was used to fit the random forest model and predict current habitat niche, with parameters for number of trees (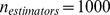), number of variables (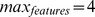), and node size (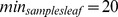) [54].

**Figure 3:**
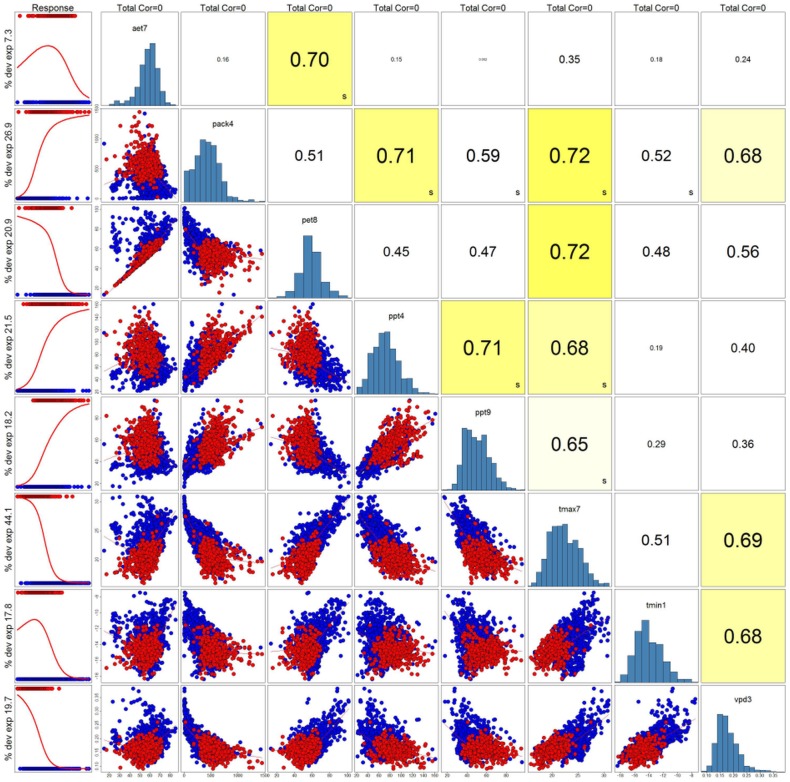
Selected predictor variables based on Principal Component Analysis and a maximum correlation filter of 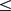
0.75. Scatter plots represent one-to-one covariate plots where red points represent *P. albicaulis* presence, and blue points represent absence from field data. Far-left columns display logistic-regression of covariates from Generalized Additive Modeling using the Software for Assisted Habitat Modeling (SAHM [59]).

First pass filtering of environmental covariates was performed using Principal Component Analysis (PCA) to generate proxy sets [55]–[57]. After initial list was constructed, an additional filter was imposed on the variables with a 0.75 maximum correlation threshold to avoid collinearity issues (Fig. 3) [55]. Physiologically relevant variables to *P. albicaulis* presence were given precidence in final culling in cases of correlation above the specified maximum threshold. The final variable list selected were tmin1, vpd3, ppt4, pack4, tmax7, aet7, pet8, ppt9 (Table 2). The Software for Assisted Habitat Modeling (SAHM) was used to visualize correlations with the pairs function embeded in the VisTrails scientific workflow management system [58], [59].

**Table 2:** Bioclimatic predictor variable list.

Code	Predictor Variable
tmin1	Minimum Temperature January
vpd3	Vapor Pressure Deficit March
ppt4	Precipitation April
pack4	Snow Water Equivalent April
tmax7	Maximum Temperature July
aet7	Actual Evapotranspiration July
pet8	Potential Evapotranspiration August
ppt9	Precipitation September

Final predictor variable set for Random Forest modeling. All variables were calculated as a 30-year climate mean from 1950–1980.

Model evaluation was performed under a variety of methods. An out-of-bag (OOB) error estimate was calculated by comparing the modeled probability of presence using approximately two-thirds of the field data, while withholding a subset of the remainder. Accuracy was evaluated by calculating: 1) the sensitivity, representing the true positive rate (TPR), 2) the specificity, representing the true negative rate (TNR), 3) the receiver operator characteristic curve (AUC). Importance of a specific predictor variable was calculated by examination of the increase in prediction error within the OOB sample when the predictor variable was permuted while others were held constant [54], [60]. The rate of prediction error with permutation of a specified variable can be interpreted as the level of dependence of presence or absence response to that variable [61].

Projections for *P. albicaulis* were computed using 30 year moving climate averages for the period from 2010–2099 for both RCP 4.5 and 8.5 climate scenarios. Changes of suitable habitat area were determined using a binary classification of expected presence and absence. Binary class assignment was made under a probability of presence threshold where the ratio of sensitivity and specificity equalled 1. This method ensured an equal ability of the model to detect presence and absence. The Kappa and True Skill Statistic (TSS) were also calculated to observe how sensitivity and specificity responded under differing probability thresholds [62]. Survey plots predicted as suitable under climatic conditions in 2010 served as a reference for projections. The presence classifications were evaluated as the amount of suitable habitat changed over time, confined within specified elevational limits. To account for the need for a minimum patch size, total number of patches and median sizes using the an eight-neighbor rule (see Text S1) for patch identification were tracked over time [63].

## Results

### Model evaluation

**Figure 4:**
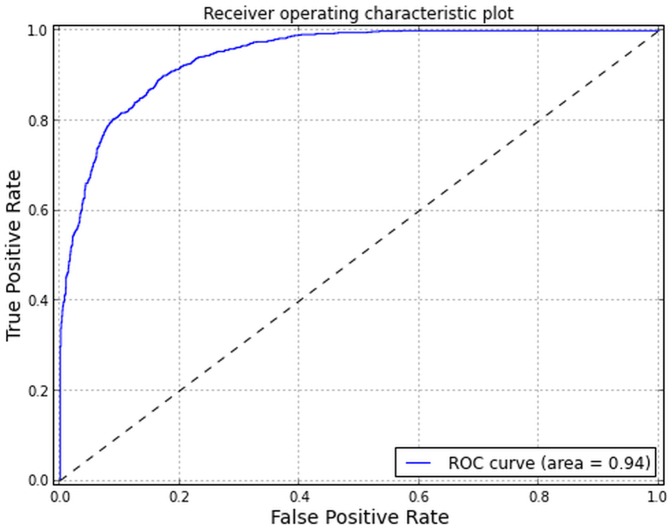
Area under curve for the receiver operating characteristic plot suggests adequate performance from the Random Forest modeling.

**Figure 5:**
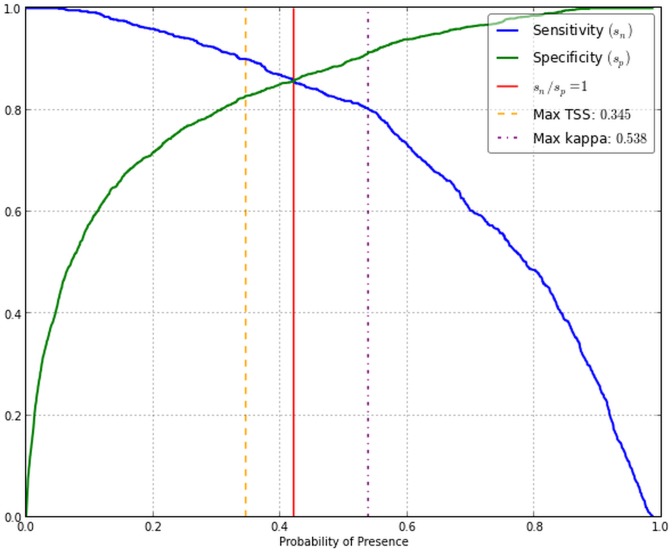
Threshold for probability of presence of 0.421 determined at the intersection of true positive rate (TPR) and true negative rate (TNR). Equivalent TPR and TNR, displayed a compromise between the maximum true skill statistic (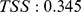) and maximum Kappa statistic (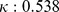).

The random forest model displayed an out-of-bag (OOB) error rate of 16.1% with greater errors of commission (13.1%) than omission (10.9%) (Table 3). The AUC was 0.94, displaying high specificity and sensitivity (Fig. 4). Threshold probability of presence for a binary classification was selected at 0.421 (i.e where sensitivity  =  specificity). A probability threshold where TPR and TNR were equal was compared to the maximum Kappa statistic (0.538) and the maximum True Skill Statistic (TSS) (0.345) and found to be a compromise between the diagnostics (Fig. 5).

**Table 3:** Confusion matrix from out-of-bag analysis.

		Validation data set	
		Presence	Absence
Model	Presence	763 *(81.9%)*	169 *(13.1%)*
	Absence	176 *(10.9%)*	1437 *(89.1%)*

Random Forest tree estimators displays higher OOB specificity than sensitivity. Area Under Curve (AUC) value of 0.94 suggests model has high predictive capacity for projecting future suitable bioclimate habitat.

**Figure 6:**
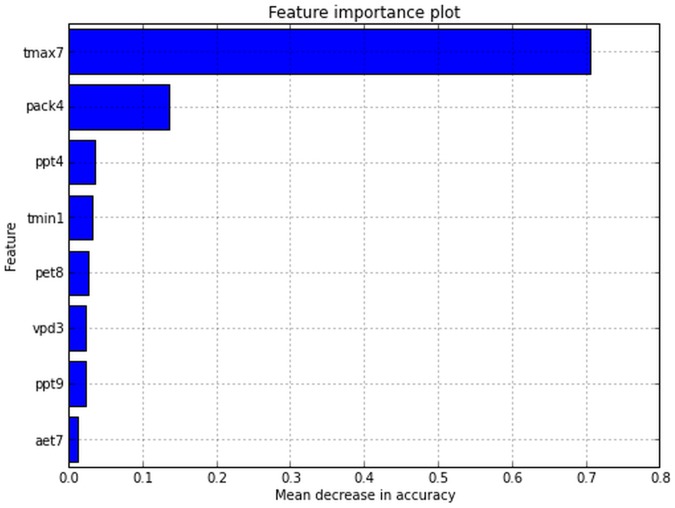
Random Forest out-of-bag variable importance plots find removal of maximum temperatures for July and April snow water equivalent to create the greatest reducing in model accuracy.

**Figure 7:**
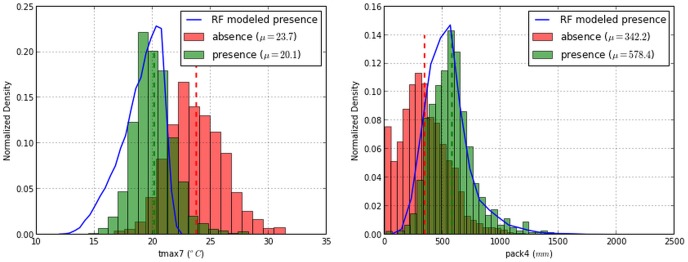
Modeled binary presence for *P.albicaulis* under 1980–2010 mean July maximum temperatures and mean April snow water equivalent bioclimate variables shows agreement with field presence data. Dotted lines designate climate means for corresponding *P. albicaulis* field points. Blue lines represent the distribution of Random Forest modeled presence within the GYA.

Estimates of variable importance plots revealed that permutation of maximum temperatures of summer months from all random trees resulted in a large drop in mean accuracy for distinguishing presence and absence of *P. albicaulis* (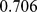 decrease in mean accuracy). This was followed by spring time snowpack (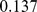 decrease in mean accuracy) (Fig. 6). Histogram plots of July maximum temperatures and April snowpack provided evidence of discrimination for presence and absence that are consistent with the modeled probability of presence for the year of calibration (Fig. 7).

**Figure 8:**
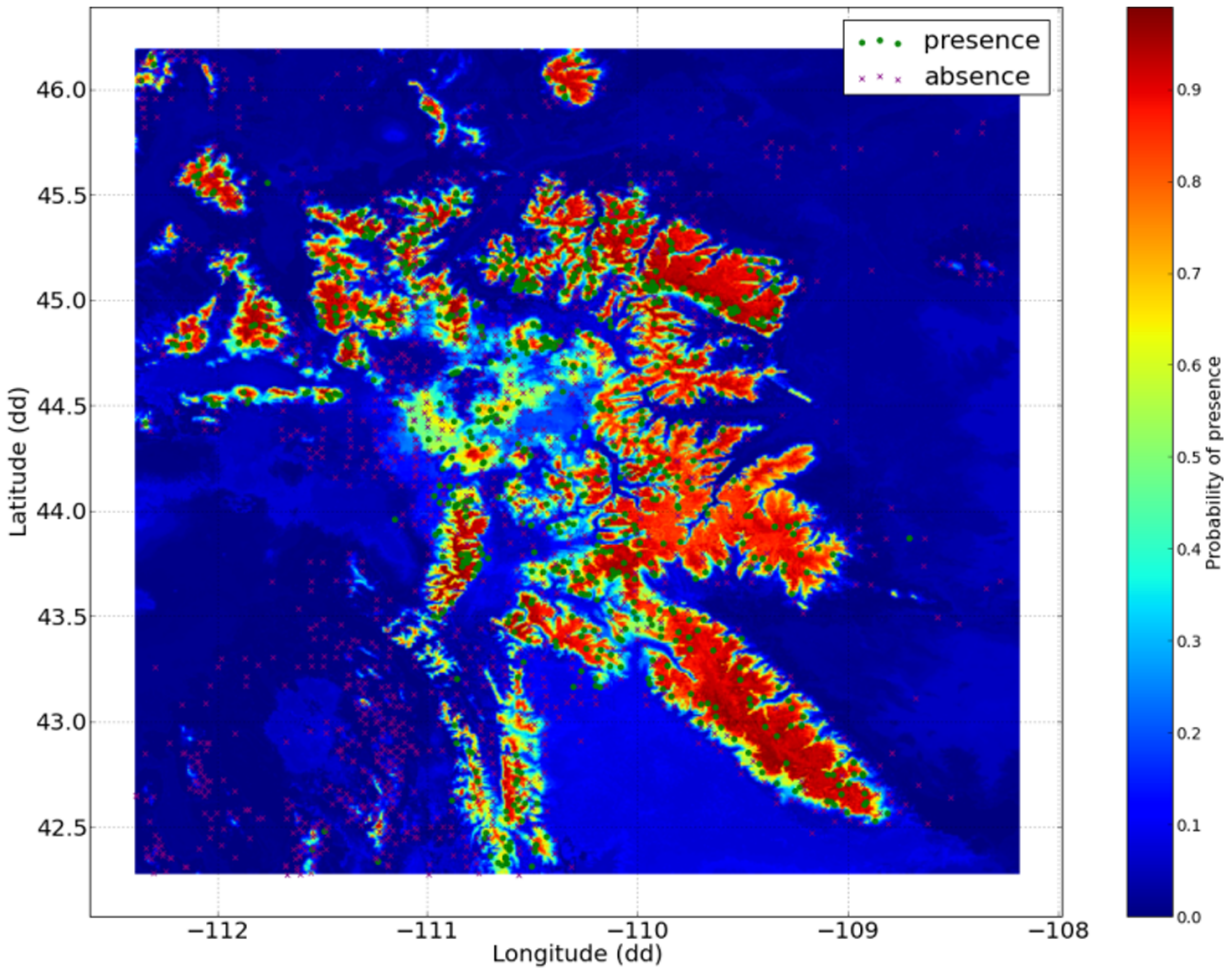
Probabiliy presence for *P.albicaulis* 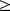
20 cm DBH within the GYA for the 2010 climate period.

Spatially explicit probability plots for the 2010 climate displayed highest probability of presence values within the 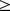
2500 m mountain ranges of the GYA in agreement with studies employing aerial imagery and remote sensing [4], [5] (Fig. 8). Assuming that the modeled suitable bioclimate for *P. albicaulis* remains similar in the next century, the model demonstrated capacity to predict probable future *P. albicaulis* suitable habitat under projected climate conditions.

### Model projections

**Figure 9:**
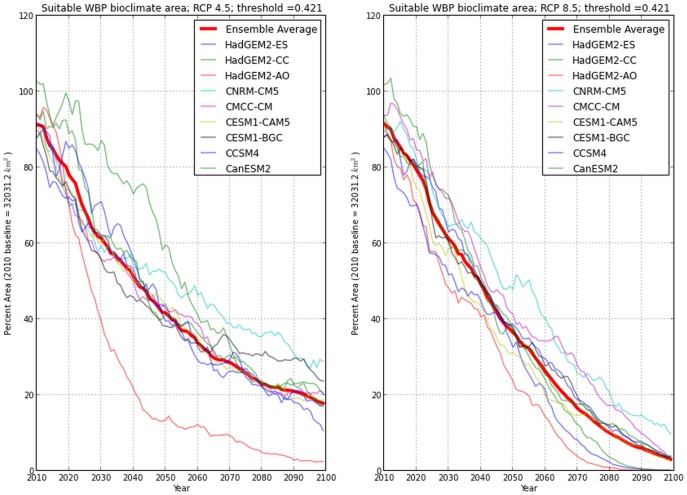
Bioclimate projections for *P.albicaulis* for 2010 to 2099 under 30-year moving averaged climates.

Under both RCP 4.5 and 8.5, there was a predicted steady reduction of suitable bioclimate habitat for *P. albicaulis* over the course of this century, with RCP 8.5 displaying steeper declines than RCP 4.5 (Fig. 9). Under the RCP 4.5 and 8.5 scenarios, suitable habitat shifts from 100–85% to 2–29% by 2099, and 100–85% to 0.04–10% by 2099 respectively (Table 4).

**Table 4:** Projected binary *P. albicaulis* presence area within GYA to 2099.

**Ensemble Average RCP 4.5**	**2010**	**2040**	**2070**	**2099**
Area (km^2^)	29250.9	16381.2	9151.1	5685.9
	*(27134*–*32858)*	*(6918*–*23359)*	*(2962*–*12477)*	*(763*–*9194)*
% Total Threshold Area*	91.3	51.1	28.6	17.8
	*(85*–*103)*	*(22*–*73)*	*(9*–*39)*	*(2*–*29)*
Mean Elevation (m)	2875.7	3020.2	3128.0	3217.9
	*(2842*–*2895)*	*(2938*–*3182)*	*(3055*–*3297)*	*(3114*–*3471)*
2.5 Percentile Elevation (m)	2356.3	2494.2	2595.0	2691.5
	*(2320*–*2376)*	*(2433*–*2656)*	*(2506*–*2758)*	*(2571*–*3041)*
97.5 Percentile Elevation (m)	3521.9	3603.5	3677.8	3734.6
	*(3507*–*3530)*	*(3551*–*3701)*	*(3636*–*3783)*	*(3673*–*3905)*
				
**Ensemble Average RCP 8.5**	**2010**	**2040**	**2070**	**2099**
Area (km^2^)	29259.3	15746.0	5271.5	960.0
	*(27188*–*32604)*	*(12985*–*19581)*	*(1247*–*8850)*	*(13*–*3105)*
% Total Threshold Area*	91.3	49.2	16.5	3.0
	*(85*–*102)*	*(40*–*61)*	*(4*–*28)*	*(0*–*10)*
Mean Elevation (m)	2874.7	3022.5	3225.5	3470.5
	*(2845*–*2893)*	*(2974*–*3061)*	*(3116*–*3412)*	*(3255*–*3749)*
2.5 Percentile Elevation (m)	2353.1	2492.2	2691.3	3001.5
	*(2322*–*2369)*	*(2436*–*2547)*	*(2553*–*2934)*	*(2622*–*3401)*
97.5 Percentile Elevation (m)	3522.1	3605.7	3739.5	3908.7
	*(3508*–*3530)*	*(3576*–*3631)*	*(3677*–*3866)*	*(3775*–*4063)*

Summary of projection outputs under RCP 4.5 and 8.5 climate scenarios displays loss of bioclimate habitat from 2010 to 2099 (low and high probability of presence GCM summaries displayed in parentheses). Projections into 2099 under all 9 GCMs suggest rapid loss of suitable bioclimate habitat to below 70% of the current modeled distribution and shifts towards the limited high elevation zones (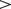
3000 m). *(*Percent threshold areas calculated from the 2010 PRISM reference probabilities of presence.*)

**Figure 10:**
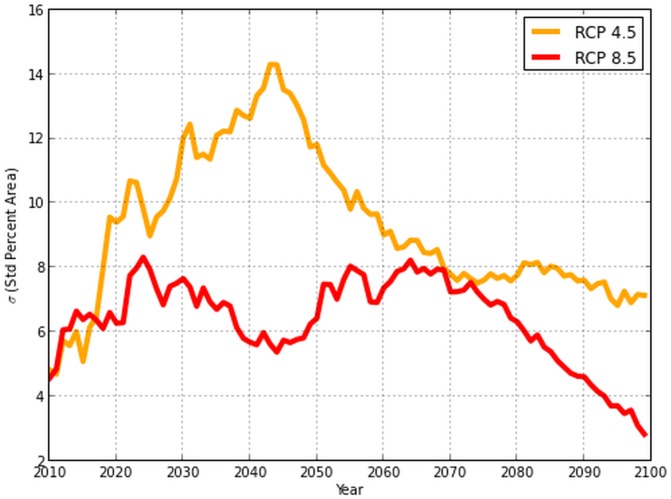
Evaluation of the standard deviation 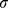 for percent suitable habitat area by RCP scenario.

CNRM-CM5, CMCC-CM, and CESM1-BGC projections showed the highest probabilities for suitable habitat area at the end of the century, while HadGEM2-AO, HadGEM2-ES, and HadGEM2-CC indicated the lowest probabilities. The standard deviations per year for both RCPs progressively decreased over time (Fig. 10). Among climate scenarios, standard deviations for both RCPs display low variability for the first five projection years and a rapid increase of variability peaking at 2043. For RCP 4.5, high variability existed primarily due to differing climate projections by models HadGEM2-AO and HadGEM2-CC, resulting in uncertainties in probabilities of presence fluctuating between 8 and 15% until 2068, after which variability was between 6–8%. Under RCP 8.5, standard deviations between GCMs were consistently lower than RCP 4.5. Regardless of the GCM, by 2079 the areas of suitable habitat converged to similar values.

**Figure 11:**
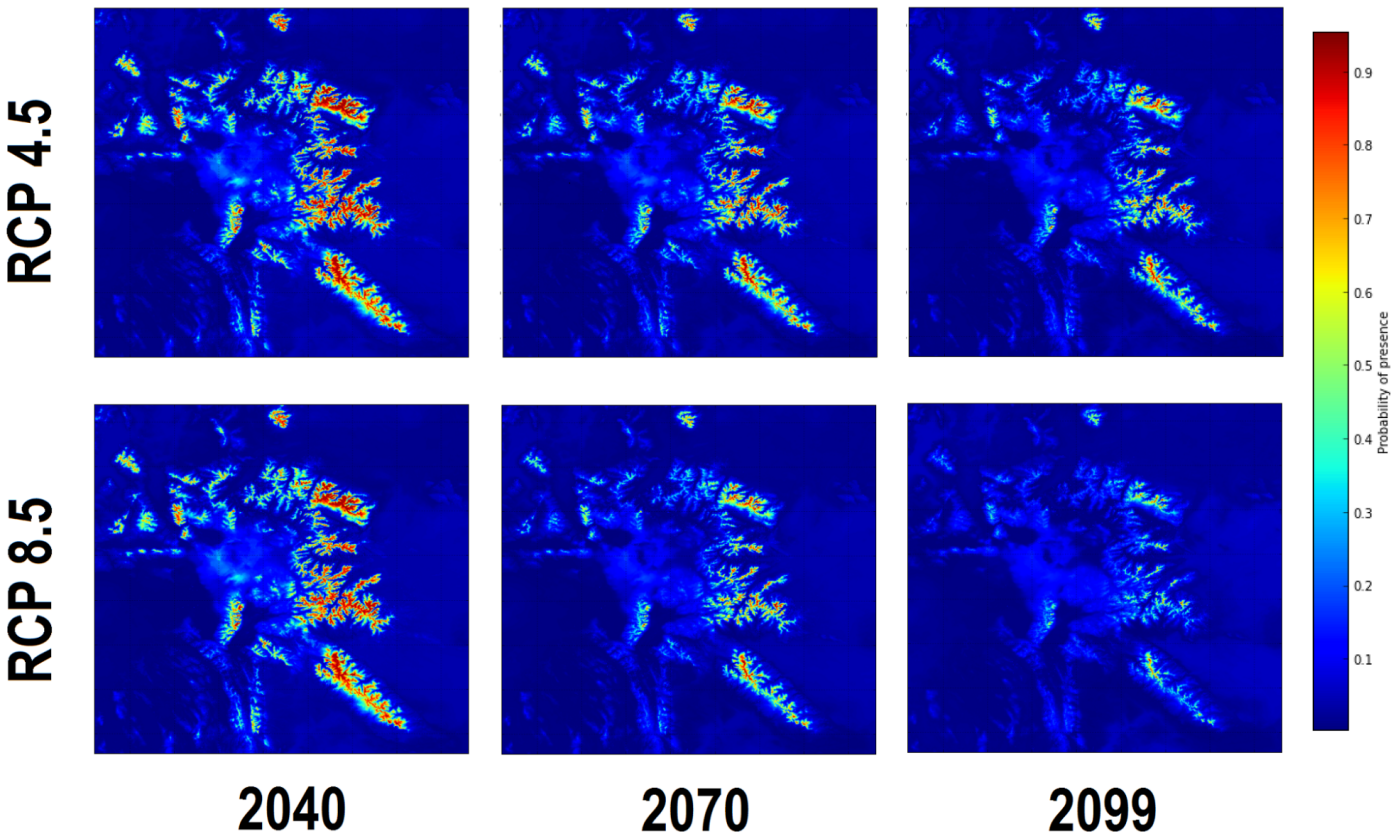
Spatially explicit probabilty surfaces for 2040 to 2099 suggest contraction of suitable bioclimatic habiatat for *P.albicaulis* into the 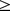
2500 m elevation zones.

Spatially explicit mapping of probability surfaces presented similar contractions of *P. albicaulis* habitat suitability toward the upper elevation zones of the GYA that included the Beartooth Plateau and Wind River Ranges (Fig. 11). This implied that rapid warming may lead to conditions outside of the *P. albicaulis* niche in lower elevation areas, and limiting the species to the alpine zones. Elevational analysis of cells within threshold presence probabilities over time observed mean elevations of suitable bioclimates shifting from 2,875 to 3,218 m and 2,875 to 3,470 m for RCP 4.5 and 8.5 respectively. By 2099, ensemble averaged GCM projections displayed over 70% loss of habitat under both scenarios.

**Figure 12:**
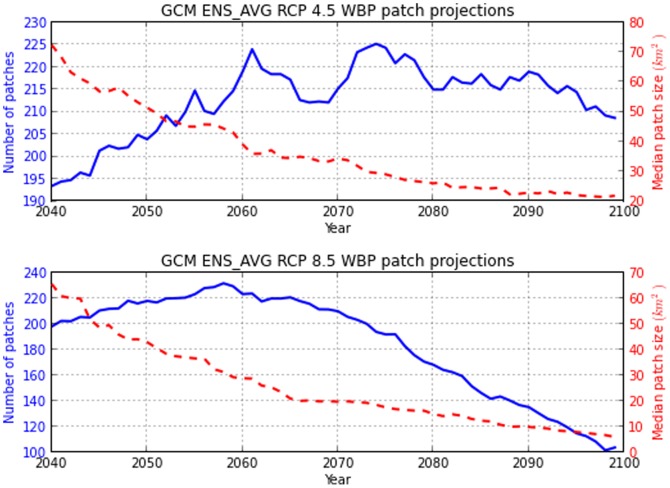
Patch dynamics of modeled *P.albicaulis*. Time series of *P.albicaulis* patch projections for number of patches and median patch size to 2099.

*P. albicaulis* patches from the 2010 baseline observed 202 patches with median patch size of 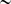
180 km^2^. Projected patch dynamics analysis denoted a quadratic relationship of patch size over time. Patch dynamics displayed a slow increase in number of *P. albicaulis* patches to a maximum at 2074 and 2057 for RCP 4.5 and 8.5 respectively, followed by a decreasing trend. RCP 4.5 patch numbers were more sporadic, displaying fluctuations across the time period compared to RCP 8.5 associated with the greater interannual climate variability amongst GCM models. Median patch size saw a steady decrease from 72–65 km^2^ to 21–8 km^2^ for RCP 4.5 and 8.5 respectively, for the projection period, suggesting habitat loss through fragmentation (Fig. 12).

## Discussion

In this analysis, the spatiotemporal patterns for *P. albicaulis* distributions were assessed under nine climate models and two emissions scenarios. Bioclimate modeling of *P. albicaulis* illustrated that presence and absence were strongly separated by summer temperatures and spring snowpack. This was in agreement with empirical findings of *P. albicaulis* presence in cool summertime environments where July temperatures range between 4–18°C [64]. Concordantly, these cool summer regions were synonymous with late snow melt, supporting snowpack as an important feature in distinguishing presence and absence.

Future projections by all nine GCMs suggested a contraction in suitable *P. albicaulis* climate area by the end of the century to 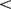
30% of current conditions. This was consistent with the results from various other research using either niche models or hybrid process models, predicting similar amounts of *P. albicaulis* contraction [8], [9], [65]. Variability among projected suitable habitat areas under differing GCMs decreased as all projected maximum temperatures increased above 1°C from the 100 year historic mean. This pattern of warming convergence occurred earlier for the GCMs under the RCP 8.5 scenario than those under RCP 4.5, resulting in the observed low variability of *P. albicaulis* suitable habitat area under RCP 8.5. Despite temperature variability remaining relatively constant amongst GCMs within a RCP, once mean annual temperatures increased beyond 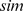 1°C from the historic average, all bioclimatic habitat models exhibited a pattern of contracting total area and variability. These results lead to the conclusion that explicit selection of a GCM to model under may not necessarily matter for *P. albicaulis* bioclimatic niche modeling studies, especially if the direction of change is solely of concern. However, if investigation of the magnitude of change is relevant, then GCM selection may directly influence the projected total suitable habitat area. This can be observed with RCP 4.5 habitat projection models differing by as much as 27% total suitable habitat area by the year 2099. Therefore arbitrary selection of a GCM for future projection modeling is likely inappropriate since it could lead to overly optimistic/pessimistic results for the species of concern.

Temporal patch dynamic analysis present an increase in fragmentation of the larger *P. albicaulis* suitable habitats over the next five decades, suggested through an increase in the total number of continuous patches but decreases in median size. This was followed by a contraction of small patches until they were almost absent from the system. Remaining habitat patches were smaller and less prevalent on the landscape by the end of the century. Reduced habitat patch size and density may reduce the likelihood for *N. columbiana* to disperse successful germinating seed caches, due to the limited size and area of suitable patch space. If changing climate habitats result in mortality within adult patches, genetic diversity may be lost resulting in a population bottleneck, thus reducing the robustness of the species to adapt to future disturbances. Experimental trials of P. albicaulis survival and fecundity under warmer and drier conditions outside the currently known range would provide greater confidence of the species ability to persist under future change. Limited analysis on seedling environmental conditions would also elucidate spatially explicit dispersal ranges and greater understanding of probable ranges for future establishment and survivorship.

Projected distributions of persistent *P. albicaulis* patches displayed a strong trend towards contraction into high elevation zones. Physiologically, there does not appear to be any upper elevation limit for *P. albicaulis* in the GYA. *P. albicaulis* in the region has been reported to survive in absolute temperatures as low as −36°C [64]. Lab experiments performed on *Pinus cembra*, a related five-needle pine residing in similar climates, were able to endure cold temperature extremes as low at −70°C without cellular tissue damage [66]. Considering the current absolute minimum temperatures the species resides in and cold tolerance of its relatives suggests that *P. albicaulis* treeline in the GYA are not limited by lower temperatures. Controlled laboratory experimentation on *P. albicaulis* tolerances to temperatures would greatly improve this physiological understanding of cold tolerance.

Elevational habitat constriction do not imply that *P. albicaulis* will be completely gone from the region, but merely the loss of suitable climate habitat. Currently pre-established adult age class individuals will likely persist, since projected conditions of increased temperatures and CO_2_ concentrations physiologically indicate increased growth rates of *P. albicaulis* [67]. Furthermore, micro-refugia sites may exist in the GYA that support *P. albicaulis* survival into the future, but were failed to have been modeled due to the coarseness of 30-arc-second climate data resolution. Since this bioclimatic envelope modeling approach was parameterized by the realized niche from in-situ data, it was difficult to determine if lower elevation limits are driven by warmer climate conditions or competition for light, water, or nutrients [15], [17]. For example, lower treeline limits for *P. albicaulis* maybe driven primarily by competitive exclusion from late seral species *A.lasiocarpa, P.contorta*, and *P.engelmannii*. This follows from paleoecological pollen records of competitor migration during the Early Holocene (9000–5000 yr B.P), when climate conditions were warmer and drier. Longer growing seasons allowing competitors to invade likely drove *P. albicaulis* communities +500 m in elevation [68]–[70]. If future climate conditions become analogous to this Early Holocene period, invasion of competitor species will likely contract *P. albicaulis* habitat to the limited high elevation zones of the GYA, specifically the Beartooth Plateau region and Wind River range [71].

## Conclusion

This analysis examined the future of *P. albicaulis* suitable climate in the GYA and explicitly addressed the question of distribution variability under 9 representative GCMs and 2 emission scenarios. Increases in temperature within the GYA will likely result in a high level of contraction of suitable climate habitat for *P. albicaulis* over the next century. This contraction was consistent for all GCM projections, with approximately 20% uncertainty in total probable area. This analysis recommends that care be taken for species distribution modeling in future studies during the selection of GCMs due to their relevance for magnitudes of change. GCM ensemble averaging may be a solution to this issue, however it should be noted that averaging should take place after an individual GCM is projected in order to maintain interannual variability.

Although other studies have examined *P. albicaulis* species distribution models [8], [65], [72], this study is a step forward through its focus on relevant regional scale design, expansive local datasets, inclusion of high resolution climate and dynamic water balance variables, and selective projection under the latest AR5 GCMs. It is reiterated that the bioclimate niche model approach has high utility for understanding habitat conditions through correlative relationships with environmental variables, however, it may fail to explicitly model competitive exclusion, disturbance, phenotypic plasticity, and other complex interactions that are vital in determining a species' actual presence as it experiences changes in climate [15], [17], [73], [74]. These unmodeled factors create uncertainties suggesting that this modeling effort does not identify the full potential climatic range of *P. albicaulis* in the future. Uncertainties also exist regarding new suitable climates that may occur outside the current species range. Despite most rangewide studies confirming our results of total suitable habitat area reduction, there is potential for previously unsuitable habitat to become available under future climate change in the Northern regions [8], [22], [65]. Caution is therefore advised to individuals interpreting these findings. Changing climate will inevitably result in impacts on biomes and community structures. As such, mitigation and adaptation for potential futures are vital to conservation of climate sensitive species [75]. Future research that combines bioclimatic niche modeling with a mechanistic based disturbance, dispersal, and competition model will likely provide greater insight to the potential range of *P. albicaulis* in a climate changing world [76], [77]. It would furthermore provide insight towards informing management options for restoration that may include controlled fire, selected thinning of competitor species, or assisted migration.

## Supporting Information

Text S1 **Description of water balance model and 8-neighbor rule**
(PDF)

## References

[1] Intergovernmental Panel on Climate Change (2007) Fourth Assessment Report: Climate Change 2007: The AR4 Synthesis Report. Geneva: IPCC.

[2] Tomback DF, Arno SF, Keane RE (2001) Whitebark pine communities: ecology and restoration. Island Press.

[3] Callaway RM (1998) Competition and facilitation on elevation gradients in subalpine forests of the Northern Rocky Mountains, USA. Oikos 82 : pp. 561–573.

[4] Macfarlane WW, Logan JA, Kern W (2012) An innovative aerial assessment of greater yellowstone ecosystem mountain pine beetle-caused whitebark pine mortality. Ecological Applications. 10.1890/11-1982.1 23634592

[5] Jewett JT, Lawrence RL, Marshall LA, Gessler PE, Powell SL, et al (2011) Spatiotemporal relationships between climate and whitebark pine mortality in the greater yellowstone ecosystem. Forest Science 57: 320–335.

[6] Logan JA, Macfarlane WW, Willcox L (2010) Whitebark pine vulnerability to climate-driven mountain pine beetle disturbance in the greater yellowstone ecosystem. Ecological Applications 20: 895–902. 20597278 10.1890/09-0655.1

[7] Logan JA, Bentz BJ (1999) Model analysis of mountain pine beetle (coleoptera: Scolytidae) seasonality. Environmental Entomology 28: 924–934.

[8] Rehfeldt GE, Crookston NL, Sáenz-Romero C, Campbell EM (2012) North American vegetation model for land-use planning in a changing climate: a solution to large classification problems. Ecological Applications 22: 119–141. 22471079 10.1890/11-0495.1

[9] Rehfeldt GE, Crookston NL, Warwell MV, Evans JS (2006) Empirical analyses of plant-climate relationships for the western United States. International Journal of Plant Sciences 167: 1123–1150.

[10] Thuiller W (2004) Patterns and uncertainties of species' range shifts under climate change. Global Change Biology 10: 2020–2027. 10.1111/gcb.12727 PMC4340562 25200514

[11] Iverson LR, Prasad AM, Matthews SN, Peters M (2008) Estimating potential habitat for 134 eastern US tree species under six climate scenarios. Forest Ecology and Management 254: 390–406.

[12] Guisan A, Theurillat JP, Kienast F (1998) Predicting the potential distribution of plant species in an alpine environment. Journal of Vegetation Science 9: 65–74.

[13] Busby J (1988) Potential impacts of climate change on Australias flora and fauna. Commonwealth Scientific and Industrial Research Organisation, Melbourne, FL, USA.

[14] Hutchinson GE (1957) Concluding remarks. Cold Spring Harbor Symposia on Quantitative Biology 22: 415–427.

[15] Austin M (2007) Species distribution models and ecological theory: a critical assessment and some possible new approaches. Ecological Modelling 200: 1–19.

[16] Austin M (2002) Spatial prediction of species distribution: an interface between ecological theory and statistical modelling. Ecological Modelling 157: 101–118.

[17] Pearson RG, Dawson TP (2003) Predicting the impacts of climate change on the distribution of species: are bioclimate envelope models useful? Global Ecology and Biogeography 12..

[18] Willis KJ, Whittaker RJ (2002) Species diversity–scale matters. Science 295: 1245–1248. 11847328 10.1126/science.1067335

[19] Araújo MB, Cabeza M, Thuiller W, Hannah L, Williams PH (2004) Would climate change drive species out of reserves? an assessment of existing reserve-selection methods. Global Change Biology 10: 1618–1626.

[20] Ferrier S (2002) Mapping spatial pattern in biodiversity for regional conservation planning: where to from here? Systematic Biology 51: 331–363. 12028736 10.1080/10635150252899806

[21] Pearce J, Lindenmayer D (1998) Bioclimatic analysis to enhance reintroduction biology of the endangered helmeted honeyeater (lichenostomus melanops cassidix) in Southeastern Australia. Restoration Ecology 6: 238–243.

[22] McLane SC, Aitken SN (2012) Whitebark pine (pinus albicaulis) assisted migration potential: testing establishment north of the species range. Ecological Applications 22: 142–153. 22471080 10.1890/11-0329.1

[23] Monahan WB, Cook T, Melton F, Connor J, Bobowski B (2013) Forecasting distributional responses of limber pine to climate change at management-relevant scales in Rocky Mountain National Park. PloS ONE 8: e83163. 24391742 10.1371/journal.pone.0083163 PMC3877015

[24] Arno SF, Hoff RJ (1989) Silvics of whitebark pine (pinus albicaulis). Intermountain Research Station GTR-INT-253.

[25] Mahalovich MF, Hipkins VD (2011) Molecular genetic variation in whitebark pine (pinus albicaulis engelm.) in the inland west. In: Keane RE, Tomback DF, Murray MP, Smith CM, editors, The future of high-elevation, five-needle white pines in Western North America: Proceedings of the High Five Symposium. 28–30 June 2010; Missoula, MT. Proceedings RMRS.

[26] Pederson GT, Gray ST, Ault T, Marsh W, Fagre DB, et al (2011) Climatic controls on the snowmelt hydrology of the Northern Rocky Mountains. Journal of Climate 24: 1666–1687.

[27] Westerling AL, Hidalgo HG, Cayan DR, Swetnam TW (2006) Warming and earlier spring increase western US forest wildfire activity. Science 313: 940–943. 16825536 10.1126/science.1128834

[28] Taylor KE, Stouffer RJ, Meehl GA (2012) An overview of CMIP5 and the experiment design. Bulletin of the American Meteorological Society 93..

[29] Hibbard KA, van Vuuren DP, Edmonds J (2011) A primer on representative concentration pathways (RCPs) and the coordination between the climate and integrated assessment modeling communities. CLIVAR Exchanges 16: 12–13.

[30] Lutz JA, van Wagtendonk JW, Franklin JF (2010) Climatic water deficit, tree species ranges, and climate change in Yosemite National Park. Journal of Biogeography 37: 936–950.

[31] Deser C, Phillips AS, Alexander MA, Smoliak BV (2014) Projecting North American climate over the next 50 years: Uncertainty due to internal variability. Journal of Climate 27: 2271–2296.

[32] Beaumont LJ, Hughes L, Pitman A (2008) Why is the choice of future climate scenarios for species distribution modelling important? Ecology Letters 11: 1135–1146. 18713269 10.1111/j.1461-0248.2008.01231.x

[33] Smith WB (2002) Forest inventory and analysis: a national inventory and monitoring program. Environmental Pollution 116: S233–S242. 11833910 10.1016/s0269-7491(01)00255-x

[34] Gibson J, Moisen G, Frescino T, Edwards TC Jr (2014) Using publicly available forest inventory data in climate-based models of tree species distribution: Examining effects of true versus altered location coordinates. Ecosystems 17: 43–53.

[35] Gesch D, Oimoen M, Greenlee S, Nelson C, Steuck M, et al (2002) The national elevation dataset. Photogrammetric engineering and remote sensing 68: 5–32.

[36] Franklin J (2009) Mapping species distributions: spatial inference and prediction. Cambridge University Press.

[37] Lockman IB, DeNitto GA, Courter A, Koski R (2007) WLIS: The whitebark-limber pine information system and what it can do for you. In: Proceedings of the conference whitebark pine: a Pacific Coast perspective. US Department of Agriculture, Forest Service, Pacific Northwest Region, Ashland, OR. Citeseer, pp. 146–147.

[38] Jean C, Shanahan E, Daley R, DeNitto G, Reinhart D, et al.. (2010) Monitoring white pine blister rust infection and mortality in whitebark pine in the Greater Yellowstone Ecosystem. Proceedings of the future of high-elevation five-needle white pines in Western North America: 28–30.

[39] Edwards TC Jr, Cutler DR, Zimmermann NE, Geiser L, Moisen GG (2006) Effects of sample survey design on the accuracy of classification tree models in species distribution models. Ecological Modelling 199: 132–141.

[40] Kadmon R, Farber O, Danin A (2003) A systematic analysis of factors affecting the performance of climatic envelope models. Ecological Applications 13: 853–867.

[41] Weaver T, Dale D (1974) Pinus albicaulis in central Montana: environment, vegetation and production. American Midland Naturalist: 222–230.

[42] Daly C, Gibson WP, Taylor GH, Johnson GL, Pasteris P (2002) A knowledge-based approach to the statistical mapping of climate. Climate Research 22: 99–113.

[43] Thornthwaite C (1948) An approach toward a rational classification of climate. Geographical Review 38: 55–94.

[44] Thornthwaite C, Mather J (1955) The water balance. Publication of Climatology 8..

[45] Dingman S (2002) Physical hydrology. Prentice Hall.

[46] National Resources Conservation Service (2014) Available: http://soildatamart.nrcs.usda.gov. Accessed 2013 Apr 3.

[47] Thrasher B, Xiong J, Wang W, Melton F, Michaelis A, et al (2013) Downscaled climate projections suitable for resource management. Eos, Transactions American Geophysical Union 94: 321–323.

[48] Rupp DE, Abatzoglou JT, Hegewisch KC, Mote PW (2013) Evaluation of CMIP5 20th century climate simulations for the Pacific Northwest USA. Journal of Geophysical Research: Atmospheres 118: 10–884.

[49] Gent PR, Danabasoglu G, Donner LJ, Holland MM, Hunke EC, et al (2011) The community climate system model version 4. Journal of Climate 24: 4973–4991.

[50] Moss RH, Babiker M, Brinkman S, Calvo E, Carter T, et al.. (2008) Towards new scenarios for analysis of emissions, climate change, impacts, and response strategies.

[51] Breiman L (2001) Random forests. Machine Learning 45: 5–32.

[52] Roberts DR, Hamann A (2012) Method selection for species distribution modelling: are temporally or spatially independent evaluations necessary? Ecography 35: 792–802.

[53] Lawrence RL, Wood SD, Sheley RL (2006) Mapping invasive plants using hyperspectral imagery and breiman cutler classifications (randomforest). Remote Sensing of Environment 100: 356–362.

[54] Pedregosa F, Varoquaux G, Gramfort A, Michel V, Thirion B, et al (2011) Scikit-learn: Machine learning in Python. Journal of Machine Learning Research 12: 2825–2830.

[55] Dormann CF, Elith J, Bacher S, Buchmann C, Carl G, et al (2013) Collinearity: a review of methods to deal with it and a simulation study evaluating their performance. Ecography 36: 027–046.

[56] Booth GD, Niccolucci MJ, Schuster EG (1994) Identifying proxy sets in multiple linear regression: an aid to better coefficient interpretation. Research paper INT.

[57] Tabachnick B, Fidell LS (1989) Using multivariate statistics, 1989. Harper Collins Tuan, PD A comment from the viewpoint of time series analysis Journal of Psychophysiology 3: 46–48.

[58] Freire J (2012) Making computations and publications reproducible with vistrails. Computing in Science & Engineering 14: 18–25.

[59] Morisette JT, Jarnevich CS, Holcombe TR, Talbert CB, Ignizio D, et al (2013) Vistrails SAHM: visualization and workflow management for species habitat modeling. Ecography 36: 129–135.

[60] Liaw A, Wiener M (2002) Classification and regression by randomforest. R news 2: 18–22.

[61] Cutler DR, Edwards TC Jr, Beard KH, Cutler A, Hess KT, et al (2007) Random forests for classification in ecology. Ecology 88: 2783–2792. 18051647 10.1890/07-0539.1

[62] Allouche O, Tsoar A, Kadmon R (2006) Assessing the accuracy of species distribution models: prevalence, kappa and the true skill statistic (tss). Journal of Applied Ecology 43: 1223–1232.

[63] Turner MG, Gardner RH, O'Neill RV (2001) Landscape ecology in theory and practice: pattern and process. Springer.

[64] Weaver T (2001) Whitebark pine and its environment. In: Tomback DF, Arno SF, Keane RE, editors, Whitebark pine communities: ecology and restoration, Washington D.C, USA: Island Press.

[65] Waring RH, Coops NC, Running SW (2011) Predicting satellite-derived patterns of large-scale disturbances in forests of the pacific northwest region in response to recent climatic variation. Remote Sensing of Environment 115: 3554–3566.

[66] Sakai A, Larcher W (1987) Frost survival of plants. Responses and adaptation to freezing stress. Springer-Verlag.

[67] Chapin III FS, Chapin MC, Matson PA, Vitousek P (2011) Principles of terrestrial ecosystem ecology. Springer.

[68] Whitlock C, Shafer SL, Marlon J (2003) The role of climate and vegetation change in shaping past and future fire regimes in the Northwestern US and the implications for ecosystem management. Forest Ecology and Management 178: 5–21.

[69] Whitlock C (1993) Postglacial vegetation and climate of Grand Teton and southern Yellowstone national parks. Ecological Monographs: 173–198.

[70] Bartlein PJ, Whitlock C, Shafer SL (1997) Future climate in the Yellowstone national park region and its potential impact on vegetation. Conservation Biology 11: 782–792.

[71] Tausch RJ, Wigand PE, Burkhardt JW (1993) Viewpoint: plant community thresholds, multiple steady states, and multiple successional pathways: legacy of the quaternary? Journal of Range Management: 439–447.

[72] Bell DM, Bradford JB, Lauenroth WK (2014) Early indicators of change: divergent climate envelopes between tree life stages imply range shifts in the western united states. Global Ecology and Biogeography 23: 168–180.

[73] Keane B, Tomback D, Davy L, Jenkins M, Applegate V (2013) Climate change and whitebark pine: Compelling reasons for restoration. Whitebark Pine Ecosystem Foundation Whitepaper.

[74] Guisan A, Thuiller W (2005) Predicting species distribution: offering more than simple habitat models. Ecology Letters 8: 993–1009. 10.1111/j.1461-0248.2005.00792.x 34517687

[75] Keane RE, Tomback DF, Aubry CA, Bower EM, Campbell CL, et al (2012) A range-wide restoration strategy for whitebark pine (pinus albicaulis): General technical report. USDA FS, Rocky Mountain Research Station RMRS-GTR-279: 108.

[76] Mathys A, Coops NC, Waring RH (2014) Soil water availability effects on the distribution of 20 tree species in Western North America. Forest Ecology and Management 313: 144–152.

[77] Morin X, Thuiller W (2009) Comparing niche-and process-based models to reduce prediction uncertainty in species range shifts under climate change. Ecology 90: 1301–1313. 19537550 10.1890/08-0134.1

