# Supplementary material for: Patterns and Variability of Projected Bioclimatic Habitat for Pinus albicaulis in the Greater Yellowstone Area
Source: PLoS One. 2014 Nov 5;9(11):e111669. doi: 10.1371/journal.pone.0111669 (PMC4221071; doi:10.1371/journal.pone.0111669)
Supplement: Text S1 — Description of water balance model and 8-neighbor rule (PDF) [file pone.0111669.s001.pdf]

Supporting Information Text S1

Water Balance Model

A simple bucket model utilizing a Thornthwaite-type equation will be applied in this study, following the methods described by Dingman [1] for water balance:

Given known climate variables, the melt factor F_m is calculated,

$$T_a \leq 0^oC : F_m = 0$$

$$0^oC < T_a < 6^oC : F_m = 0.167T_a$$

$$T_a \leq 6^oC : F_m = 1$$

where T_a is the mean monthly temperature (^oC). This allows the modeling the amount of rain and snow from the known P_m precipitation values (mm):

$$RAIN_m = F_m P_m$$

$$SNOW_m = (1 - F_m)P_m$$

From these the monthly total accumulated snow pack $PACK_m$ and snow melt $MELT_m$ can be determined:

$$PACK_m = (1 - F_m)^2 P_m + = (1 - F_m)PACK_{m-1}$$

$$MELT_m = F_m(SNOW_m + PACK_{m-1})$$

The monthly water input W_m to the system (or individual cell) is:

$$W_m = RAIN_m + MELT_m$$

Values of monthly water input are related to the maximum water holding capacity (WHC) from empirical interpolation $SOIL_{max}$, and the potential and actual evapotraspiration (PET_m) & (AET_m), as follows:

$$SOIL_m = minimum[SOIL_{max}, [(W_m - PET_m) + SOIL_{m-1}]]$$

Using the heat load (HL) equation from McCune and Keon [2], reproduced here:

$$A_f = |180 - |aspect - 225||$$

$$HL = 0.339 + 0808[cos(L)cos(S)] - 0.196[sin(L)sin(S)] - 0.482[cos(A_f)sin(S)]$$

where L is latitude, S is slope, A_f is the folded aspect. PET_m can be derived

$$PET_m = 29.8 * n_{days} * DL_m * \frac{e_a(T_a)}{T_a + 273.2} * HL$$

where, e_a is the saturation vapour pressure at T_a from Campbell and Norman [3]:

$$e_a = 0.611e^{(\frac{17.502T_a}{T_a+240.97})}$$

additionally, vapor pressure deficit can be determined by calculating the actual vapor pressure using $T_{dewpoint}$ for the temperature variable and subtracting actual vapor pressure from saturation.

n_{days} = days in the month, and DL_m is the daylength from the 15th day of the month defined by:

$$DL_m = \frac{2cos^{-1}[-tan(\delta_m)tan(\lambda)]}{\omega}$$

for δ_m as the solar declination angle, λ is latitude, and ω is the angular velocity of the Earth's rotation ($0.2618\frac{rad}{hr}$).

AET_m can be derived as the smaller of either the sum ($\Delta_{SOIL} + W_m$) or PET_m, where Δ_{SOIL} is defined as:

$$\Delta_{SOIL} = SOIL_{m-1} - SOIL_m = SOIL_{m-1} * [1 - e^{-\frac{(PET_m - W_m)}{SOIL_{max}}}]$$

Provided this water balance model and mean monthly climate for the temporal scale of interest, this simple working model of climate and water/energy balance presents a set of predictor variables for vegetation distribution modeling.

Eight Neighbor Rule

One common method of determining connectivity of a local population patch is through adjacently of common pixel classes. This assumes that a local population patch can be qualitified, only if it consists of 2 or more adjacent pixels of the same 'presence classification'. Connectivity for a pixel is determined by either sharing a complete side (4-neighbor rule) or both complete sides and diagonal adjacency (8-neighbor rule) of another neighboring pixel [4]. For this study an implementation of the 8-neighbor rule is used to not overly restrict the definition of a local population patch. The implementation is provided below in Python script using the SciPy scientific computing library [5].

```
{
#Author:  Tony Chang
#Abstract:  Functions to determine patch size and number under
# the 8 or 4 neighbor rule, using the SciPy scientific computing library
#Dependencies: scipy and numpy

import numpy as np
import scipy as sp
from scipy import ndimage

def get_patch(mat, method = 8):
    if method ==8: #default 8 neighbor cell method
        s = sp.ndimage.generate_binary_structure(2,2)
        labeled_array, numpatches = sp.ndimage.label(mat, s)
    else: #4 neighbor cell method
        labeled_array, numpatches = sp.ndimage.label(mat)
        sizes = sp.ndimage.sum(mat, labeled_array, range(1, numpatches+1))
     return(labeled_array, numpatches, sizes)

def extract_edges(mat, size):
    edge = sp.ndimage.distance_transform_edt(mat==0) ==1
    if (size > 1):
        s = sp.ndimage.generate_binary_structure(2,1)
        edge = sp.ndimage.binary_dilation(edge, s, iterations=size-1)
     return(edge)
}
```

References

1. Dingman S (2002) Physical hydrology. Prentice Hall.

2. McCune B, Keon D (2002) Equations for potential annual direct incident radiation and heat load. Journal of Vegetation Science 13: 603–606.

3. Campbell GS, Norman JM (1998) An introduction to environmental biophysics. Springer.

4. Turner MG, Gardner RH, O'Neill RV (2001) Landscape ecology in theory and practice: pattern and process. Springer.

5. Jones E, Oliphant T, Peterson P, van der Walt S, Colbert C, et al. (2001). SciPy: Open source scientific tools for Python. URL `http://www.scipy.org/`. Accessed 2014-04-16.
